# Herpes simplex virus type 2 infection triggers AP-1 transcription activity through TLR4 signaling in genital epithelial cells

**DOI:** 10.1186/s12985-018-1087-3

**Published:** 2018-11-12

**Authors:** Xiaowen Lv, Huanru Wang, Airong Su, Shijie Xu, Ying Chu

**Affiliations:** 1 Department of Paediatrics, Affiliated Hangzhou First People’s Hospital, Zhejiang University School of Medicine, 261# Huansha Road, Hangzhou, 310006 China; 2 Center for Public Health Research, Medical School, Nanjing University, 22# Hankou Road, Nanjing, 210098 China; 3 Central Laboratory, The Second Affiliated Hospital of Nanjing Medical University, 121# Jiangjiayuan, Nanjing, 210029 China; 4 Central Laboratory, The Affiliated Wujin People’s Hospital of Jiangsu University, #2 North Yongning Road, Changzhou, 213002 China

**Keywords:** Herpes simplex virus type 2 (HSV-2), Activator protein 1 (AP-1), Toll-like receptor 4 (TLR4), Genital epithelial cell

## Abstract

**Background:**

The pattern recognition receptors (PPRs) are the earliest phase of the host defense against pathogens in genital epithelium, and toll-like receptors (TLRs) are best characterized PPRs mediating innate immune responses. Herpes simplex virus type 2 (HSV-2), a member of *herpesviridae* family, causes one of the most prevalent sexually transmitted infection in the world. In this paper, we described that HSV-2 infection would induce activator protein 1 (AP-1) via TLR4-MyD88/TRIF pathway in human genital epithelial cell.

**Methods:**

TLRs expression profiles and changes was investigated in HSV-2-infected cells. The effect of TLR4-MyD88/TRIF on HSV-2-induced AP-1 activation and viral replication was also evaluated. The TLR4 translocation change was examined after viral infection. Finally, viral ICP0 effect on TLR4 signaling and TLR4-promoter regulation were primarily studied.

**Results:**

HSV-2-induced AP-1 activation was dependent on TLR4 and downstream adaptor molecules MyD88 and TRIF. And also, TLR4, MyD88 and TRIF was proved to affect HSV-2 replication. AP-1 activation would also be enhanced via overexpression of myeloid differentiation protein 2 (MD2), implicating that it might be a necessary accessory for TLR4 to sense HSV-2 infection. Protein quantification of cytoplasmic and membrane-associated TLR4 revealed that HSV-2 infection increased membrane-anchoring TLR4 level, but not cytoplasmic ones. Viral ICP0 could augment cellular AP-1, TLR4 promoter activation and TLR4 expression level. The specific inhibitor treatment and transcription factor binding site scanning in TLR4 promoter region showed that AP-1 activity was essential for TLR4-promoter activation.

**Conclusions:**

Taken together, HSV-2 infection could stimulate AP-1 activation via TLR4-MyD88/TRIF axis, and then feedback to up-regulate TLR4 expression in human genital epithelial cells.

## Background

Herpes simplex virus type 2 (HSV-2), a member of *herpesviridae* family, is one of the most prevalent human pathogens in the world, which causes genital herpes and can be transmitted to central nervous system (CNS) to establish lifelong infection [1]. HSV-2 is primarily transmitted through sexual contact and is common among persons infected with HIV-1 [2, 3]. In the Americas and Europe, HSV-2 seroprevalence is 50% among HIV-1 infected men who have sex with men [4]. It is well established that HSV-2 infection facilitated the perseverance of HIV-1 epidemic [5]. Also, HSV-2 infection is an important bacterial vaginosis risk factor, thus it may co-infect with other bacterial pathogen in clinical [6]. However, until now, there are no effective medicines or preventive vaccine for genital herpes.

The human genital mucosa is an important tissue structure for innate immune systems and is the natural barrier to defense against sexually transmitted pathogens [7]. Due to the compactness of epithelial cells and their cell-cell tight junctions, genital epithelium could defend against most of pathogens via physical blocking. Certain pathogens are evolving to disrupt epithelium to establish primary infection. For host defense system, mucosal epithelial cells could constitutively express immune-associated molecules to inhibit infection or sense them to activate local inflammation to recruit immune cells. A set of pattern recognition receptors (PPRs) were found to be expressed in genital epithelial cells, which was proven to recognize microorganisms or their associated components, and stimulate downstream anti-microbial immune responses. Toll-like receptors (TLRs), which commonly express on a range of immune cells and epithelial cells, represents an essential components for cellular innate immunity [8, 9]. There are several published manuscripts reporting the interaction of TLRs and pathogens, and TLRs-mediated downstream anti-microbial activities. Derbigny et al. reported that *Chlamydia* induced IFN-β synthesis in infected murine oviduct epithelial cells to modulate the adaptive immune responses via TLR3 [10]. Nazli et al. demonstrated that HIV-1 envelope glycoprotein gp120 could induce NF-κB activation via TLR2 and TLR4 in human female genital epithelium, which might activate innate immune in reproductive tract [11]. Another described that natural ligands of TLRs would induce antiviral responses against HSV-2 infection in genital epithelial cells [12]. Evidently, TLRs-associated signaling activation would sometimes enhance innate immune response and eliminate infection, but in some cases, pathogens would utilize host TLRs-associated responses to facilitate its life cycle to establish persistent infection.

Many published manuscripts related to the studies of the interaction of TLRs and HSV, and reported that TLR2 and TLR9 were involved in innate antiviral responses [13–16]. However, the infection models used in these studies was central neuronal cells, immune-competent cells or transgenic mice models, which were totally distinct with mucosal epithelial cells. Liu et al. firstly reported the association between TLR4-NF-kB pathway and HSV-2 infection in human cervical epithelial cells [17]. Our previous studies described that HSV-2 infection could stimulate mitogen-activated protein (MAP) kinase pathway and enhance AP-1 activation, and AP-1 activation was essential for effective viral replication [18]. However, less studies was related to the relationship between MAPK pathway and TLR4 in HSV-2 infected genital epithelial cells. In this study, TLRs expression profiles and changes after HSV-2 infection was evaluated in human genital epithelial cells, and the relationship between TLR4 and AP-1 activation was investigated. Our finding revealed that TLR4 might play a role in HSV-2 sensing and take part in viral life cycle in human genital epithelium.

## Methods

### Reagents, cell lines, plasmids, viruses

Lipopolysaccharide (LPS) was purchased from Sigma-Aldrich (St. Louis, MO, USA). Acyclovir was obtained from National Institutes for Food and Drug Control in China (Beijing, China). Dual-Glo luciferase assay kit was obtained from Promega Bio-technology (Madison, WI, USA). Odyssey blocking buffer, IRDye 680 and IRDye 800 secondary antibodies were obtained from LI-COR (Lincoln, NE, USA). Anti-human TLR4 (sc-293,072), anti-GAPDH (sc-32233), anti-HSV-2 gD (sc-56988) and anti-β-actin (sc-69,879) were purchased from Santa Cruz (Santa Cruz, CA, USA). Anti-c-Jun (#2315), anti-p-c-Jun (#2361) and anti-MEK1/2 (#8727) antibodies were from Cell Signaling Technology (Beverly, MA, USA). Anti-GFP (TA-06) was from ZSGB-Bio (Beijing, China). DRAQ5 was from Thermo Fisher Scientific (Grand Island, NY, USA).

VK2/E6E7, Ect1/E6E7, Endo1/E6E7 and HEC-1-A cells were obtained from American Type Culture Collection (ATCC, Manassas, VA, USA). HEK-293 T, Vero and U937 cells were from the Type Culture Collection of the Chinese Academy of Sciences (Shanghai, China). U937 cells were grown in RPMI-1640 medium supplemented with 10% fetal bovine serum (FBS) and 1 mM HEPES. HEK-293 T was maintained in Dulbecco’s modified Eagle’s medium (DMEM) with 10% FBS. VK2/E6E7, Ect1/E6E7 and Endo1/E6E7 were established from the normal vaginal mucosal tissue transformed by HPV-16 E6/E7, and would be maintained in keratinocyte-serum free medium with 0.1 ng/ml human recombinant EGF, 0.05 mg/ml bovine pituitary extract, and additional with 0.4 mM calcium chloride. HEC-1-A cells were grown in McCoy’s 5a medium with 10% FBS. Vero-ICP10P, an HSV-2 infection indicator cell line, was generated from Vero cells stably-transfected with HSV-2 ICP10 promoter-driven luciferase reporter plasmid. Cells mentioned in this paper were maintained at 37 °C in a 5% CO_2_ atmosphere. All base medium and FBS were purchase from Thermo Fisher Scientific.

AP-1-luc reportor plasmids (pAP-1-luc) were from Clontech (Mountain View, CA, USA). pRL-TK *Renilla* luciferase control vectors were obtained from Promega Bio-technology. pFlag-CMV1-hMD2 (Plasmid #13028) was obtained from Addgene (Cambridge, MA, USA). pcDNA3-ICP0(2)-GFP were kindly gifted from Dr. Claus-Henning Nagel, Heinrich Pette Institute, Leibniz Institute for Experimental Virology. pcDNA3.1-hTLR4 plasmid was constructed via cloning full-length TLR4 coding sequence from U937 cells cDNA library, and then inserting into pcDNA3.1 (Thermo Fisher Scientific). pGL4-TLR4-promoter was constructed by amplifying a 1100-bps TLR4 promoter fragment (− 801/+ 299) and then cloning into pGL4.17 vector (Promega). Truncated TLR4-promoter luciferase reporter plasmids were constructed via subcloning truncated regions of promoter (− 385~ + 299, − 220~ + 299 and − 75~ + 299) into pGL4.17.

HSV-2 (G) strain was kindly gifted from Dr. Erguang Li, Medical School, Nanjing University, China, and was propagated on HEK-293 T cells and titrated on Vero cells as described previously [19].

### Western blot and in-cell Western

Cells were washed once with pre-cold phosphate buffer saline (PBS) and then lysed on ice using RIPA lysis buffer (Santa Cruz). Lysate was centrifuged at 12,000×g, 4 °C for collection of the supernatants for total protein extraction. Membrane-associated and cytoplasm-associated proteins were extracted using membrane/cytoplasmic protein extraction kit (Sangon, Shanghai, China). The protein concentrations were determined using BCA protein assay kit (Thermo Fisher Scientific). After separated via SDS-PAGE, the proteins were transferred to polyvinylidene difluoride (PVDF) membranes (Millipore, Billerica, MA, USA). The membranes were blocked with Odyssey blocking buffer and then inoculated in primary antibodies at room temperature (RT) for 1 h. After 5 times wash with PBS-T buffer (PBS supplemented with 0.1% Tween-20), the blots were incubated in IRDye IgG with 1:10,000 dilutions for 1 h. The membranes were visualized under LI-COR Odyssey Infrared Imager (LI-COR), and the band density could be determined via Odyssey software.

In-cell Western was performed in 96-well plate. The cells cultured in a 96-well plate were fixed with 4% paraformaldehyde for 20 min at RT and permeabilized by 5 washes in PBS-0.1% Triton-X 100 with 5 min for each wash. The monolayers were blocked for 90 min in blocking buffer (4% non-fat dry milk) and then incubated with primary antibodies diluted in blocking buffer (1:200) for 2 h at RT. After washing with PBS-T buffer, the cell layers were stained with IRDye IgG (1:1500) for 1 h, rinsed and scanned in Odyssey Infrared Imager. Relative protein expression level was normalized against DRAQ5 staining (nucleus DNA staining).

### Luciferase assay

HEC-1-A cells were seeded into 96-well plates at a density of 2.5 × 10^4^ cells per well. When reaching 90% confluency, cells were transiently transfected with 25 ng pAP-1-luc or promoter reporter plasmids and 5 ng pRL-TK per well or co-transfected with 25 ng pAP-1-luc, 5 ng pRL-TK and 70 ng transient expression plasmids using Lipofectamine 2000 reagent (Thermo Fisher Scientific) as manufacturer’s instruction. Cells were then cultured for 24 h and then treated as described. The luminescence was determined by GloMax-96 Microplate Luminometer (Promega). The results are shown as means ± SD of triplicate wells and expressed as relative luminescence units (RLUs).

### RNA extraction, PCR and real-time PCR

Total RNA was extracted using TRIzol reagent (Thermo Fisher Scientific) as manufacturer’s instructions. Complementary DNA (cDNA) was reverse-transcribed in a 20-μl volume using ReverTra Ace qPCR RT kit (TOYOBO, Osaka, Japan). PCR amplification was performed using TaKaRa Ex Taq DNA polymerase (TaKaRa, Shiga, Japan), and the thermo cycling protocol was as follows: 95 °C for 5 min, 25 cycles of denaturation at 95 °C for 45 s, annealing at 60 °C for 45 s, and extension at 72 °C for 1 min. Real-time PCR was performed in triplicate on ABI Prism 7300 Sequence Detection System using the SYBR Green PCR Master Mix (TOYOBO) according to the manufacturer’s protocols. Message RNA transcriptions were standardized against housekeeping gene GAPDH. All Primers used in this study were referred from RTPrimerDB (http://www.rtprimerdb.org/), verified in-house and listed in Table 1.

**Table 1:** Real-time PCR primers sequences

*TLR1*	Sense: CAGTGTCTGGTACACGCATGGT;
	Antisense: TTTCAAAAACCGTGTCTGTTAAGAGA;
*TLR2*	Sense: GCCTCTCCAAGGAAGAATCC;
	Antisense: TCCTGTTGTTGGACAGGTCA;
*TLR3*	Sense: TGGTTGGGCCACCTAGAAGTA;
	Antisense: TCTCCATTCCTGGCCTGTG;
*TLR4*	Sense: AAGCCGAAAGGTGATTGTTG;
	Antisense: CTGAGCAGGGTCTTCTCCAC;
*TLR5*	Sense: TGCCTTGAAGCCTTCAGTTATG;
	Antisense: CCAACCACCACCATGATGAG;
*TLR6*	Sense: GAAGAAGAACAACCCTTTAGGATAGC;
	Antisense: AGGCAAACAAAATGGAAGCTT;
*TLR7*	Sense: TTTACCTGGATGGAAACCAGCTA;
	Antisense: TCAAGGCTGAGAAGCTGTAAGCTA;
*TLR8*	Sense: TTATGTGTTCCAGGAACTCAGAGAA;
	Antisense: TAATACCCAAGTTGATAGTCGATAAGTTTG;
*TLR9*	Sense: GGACCTCTGGTACTGCTTCCA;
	Antisense: AAGCTCGTTGTACACCCAGTCT;
*MD2*	Sense: CCGATGCAAGTATTTCATACACCTACT;
	Antisense: CTCCTTGGAATGTAGAAAATGTGC;
*GAPDH*	Sense: TGCACCACCAACTGCTTAGC;
	Antisense: GGCATGGACTGTGGTCATGAG;

### Small interfering RNAs (siRNAs) transient transfection

Validated siRNAs targeting human TLR4, MyD88, TRIF and negative control were purchased from Santa Cruz. HEC-1-A cells were seeded into 96-well plate at a density of 10^4^ per well, and then cultured for 24 h. When the cell confluency reached to ~ 40%, siRNAs were transfected alone (20 pmol siRNA) or co-transfected with luciferase reporter and internal control plasmids (20 pmol siRNA, 25 ng luciferase reporter plasmids and 5 ng pRL-TK) into target cells via lipofectamine 2000 reagent according to the manufacturer’s manuals. The siRNA knockdown efficiency was determined by reverse transcription PCR (RT-PCR). Cells were then treated as described, and relative luminescence units were determined as described above.

### Statistics

Statistical analysis was performed using two-tailed student t-test. Statistical significance: * *p* < 0.05, ** *p* < 0.01.

## Results

### The profiles of TLRs expression and changes after HSV-2 infection in the human genital epithelial cells

**Fig. 1:**
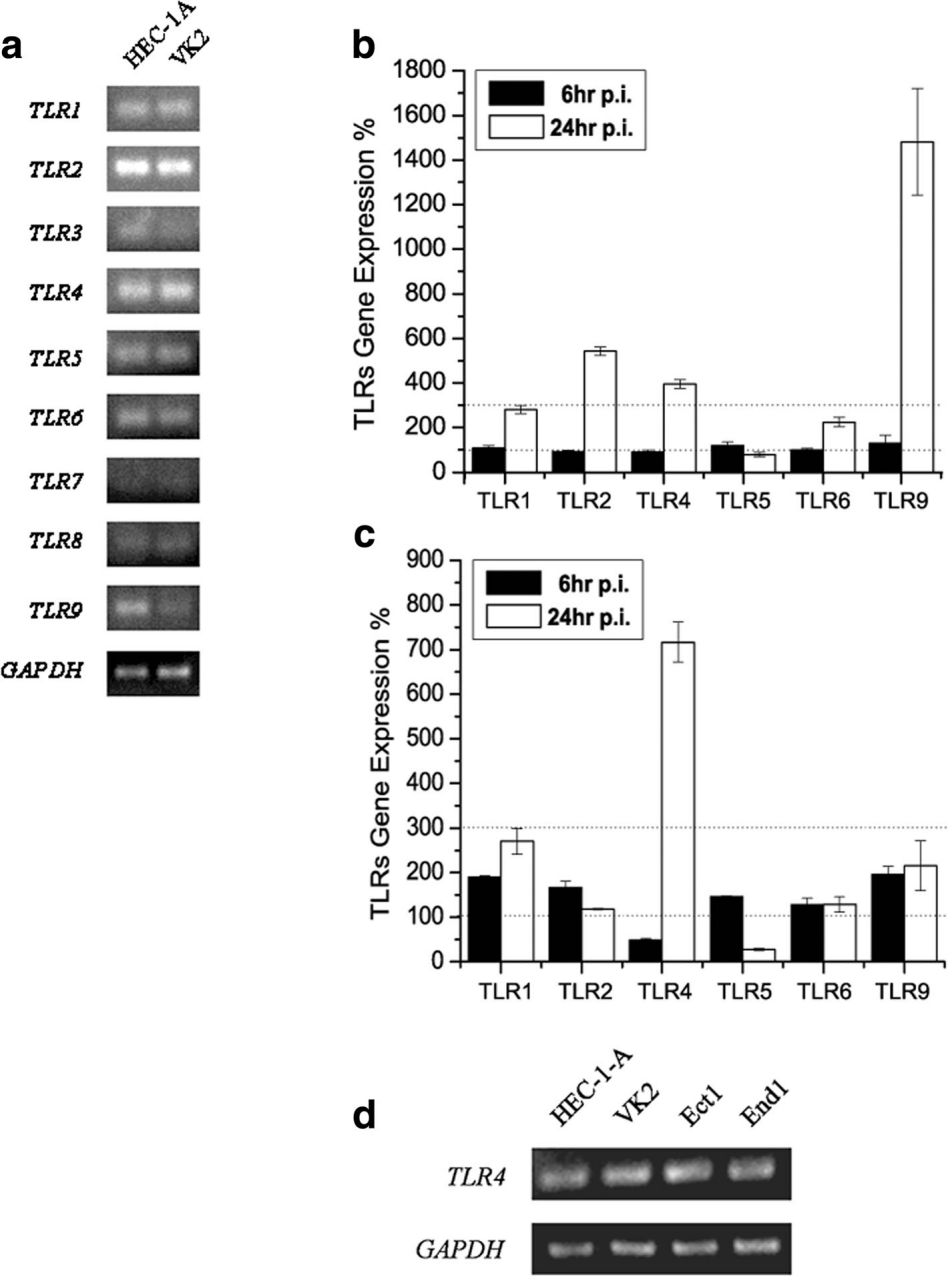
The profiles of TLRs expression in the human genital epithelial cells and their expression level regulated by HSV-2 infection. **a** TLRs expression profiles in HEC-1-A and VK2 cells. Total RNA was extracted from HEC-1-A or VK2 cells, and TLRs were detected via RT-PCR. **b**–**c** certain TLRs expression fold change after HSV-2 infection in HEC-1-A cells (**b**) and VK2 (**c**). Cells were harvested 6 h or 24 h p.i. and total RNA was extracted as described. TLRs expression was detected via real-time PCR. **d** TLR4 mRNA expression in HEC-1-A and 3 normal human genital epithelial cells with different anatomical positions. Total RNA were extracted from the different cell lines as described, and TLR4 expression were evaluated via RT-PCR. All experiments were performed three times and the representative experiment results were shown

Two kinds of typical human genital cell lines, HEC-1-A and VK2 were mainly employed and TLRs expression profiles were investigated via RT-PCR. As shown in Fig. 1a, TLR1, TLR2 and TLR4 mRNAs were highly expressing in both two cell lines, and TLR5 and TLR6 were moderate in mRNA transcription level. TLR3, TLR7 and TLR8 mRNA transcription were weak in both of cell lines. The mRNA level of TLR9 was distinct in these two cell lines, with moderate in HEC-1-A cells but weak in VK2/E6E7. After TLRs expression profiling in human genital epithelial cells, we considered whether these TLRs gene expression would be regulated by HSV-2 infection. In this study, we focused on TLR1, TLR2, TLR4, TLR5, TLR6 and TLR9 expression fold changes in two genital epithelial cells (HEC-1-A and VK2) after viral infection. The mRNA change threshold is set to 300%. The results illustrated that TLR2, TLR4 and TLR9 were up-regulated by over 300% in HEC-1-A cell 24 h post-infection (p.i.) (Fig. 1b), and only TLR4 transcription was enhanced by over 700% in VK2 cells (Fig. 1c). Although these two cell lines were with different genetic background, TLR4 expression was up-regulated after HSV-2 infection in both HEC-1-A and VK2/E6E7 cells. So we further focused on TLR4 expression and its function in genital epithelial cells in HSV-2 infection.

We also investigated the TLR4 expression in normal human genital epithelial cells comprehensively. VK2/E6E7, Ect1/E6E7 and End1/E6E7 represented normal vaginal mucosal, ectocervical and endocervical epithelial cell lines, respectively. As shown in Fig. 1d, all of the genital epithelial cell lines from different anatomical positions constitutively expressed TLR4 mRNA. We hypothesized that TLR4 might play a role in epithelial cell response to pathogens infection, and further studies would be focused on the relationship between TLR4 and HSV-2 infection in human genital epithelial cells.

### HSV-2 infection up-regulated TLR4 expression in genital epithelial cells

**Fig. 2:**
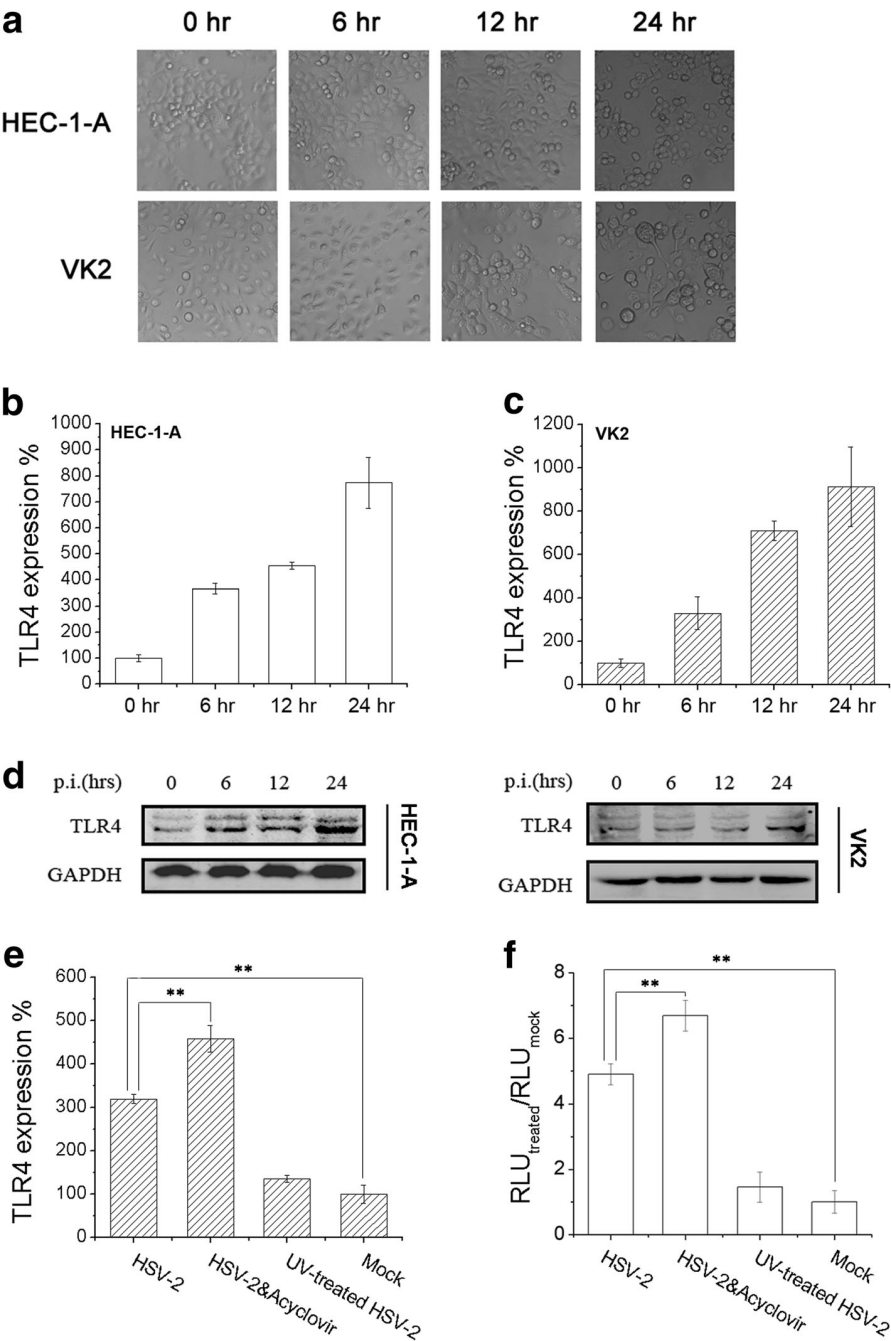
HSV-2 infection up-regulated TLR4 expression in genital epithelial cells. **a** Significant CPE was observed under optical microscope with time-dependent manner. **b**–**c** HSV-2 infection up-regulated TLR4 expression in mRNA level in HEC-1-A (**b**) and VK2 cells (**c**). Total RNA was extracted from HEC-1-A or VK2 cells at time points as described. TLR4 mRNA transcription level was determined through real-time PCR. **d** HSV-2 infection up-regulated TLR4 expression in protein level in HEC-1-A and VK2 cells. Cells were harvested at time points as described and TLR4 expression was detected via western blot. **e**–**f** Certain IE and E gene products might be the trigger for TLR4 expression up-regulation. **e** HEC-1-A cells were mock-treated or treated with acyclovir (5 μg/ml), and then infected with HSV-2. The other cell sample was infected with UV-treated HSV-2. TLR4 expression was determined via real-time PCR. **f** HEC-1-A cells were transfected with TLR4-promoter-luciferase plasmids and pRL-TK as a transfection control. After 24 h post-transfection, cells were treated as same as mentioned in (**e**). RLUs were determined as described. All experiments were performed three times. The representative experiments were shown

Investigated above showed that HSV-2 infection augment TLR4 transcription level in HEC-1-A and VK2 cells. To validate these results, we studied the TLR4 expression fold change at different time-points during HSV-2 infection. As shown in Fig. 2 a-b, HSV-2-infection enhance TLR4 mRNA transcription in a time-dependent manner in both cell lines. In Fig. 2c, HSV-2 infection could cause significant cytopathic effect (CPE) with time-dependent manner. We also determined the effect of viral infection on TLR4 expression change via western blot, and illustrated that HSV-2 infection could up-regulate TLR4 expression in protein level (Fig. 2d). Then, we primarily considered which steps of viral life cycles were essential for TLR4 expression up-regulating in genital epithelial cells. Acyclovir was a typical inhibitor against herpes viral DNA polymerases, and it could suppress viral productive cycle, but no or less influence on initial viral immediate-early (IE) or certain early (E) genes expression. UV treatment could destroy viral DNA structure and the virions would be not persistently infectious, but UV-treated virions still retain their ability to adhere to host cells or enter into them. The results was shown in Fig. 2e–f, illustrating that UV treatment could impede TLR4 up-regulation completely. But by contrast, acyclovir treatment would enhance TLR4 mRNA transcription activity significantly, which compared with that of HSV-2-infected HEC-1-A cells. Similar results were obtained via detecting TLR4-promoter activity with the same treatments. It was concluded that certain IE and certain E genes products might accumulate after acyclovir treatment, and thus activate TLR4 promoter activity and up-regulate its expression.

### HSV-2 triggered AP-1 activation via TLR4-MyD88/TRIF pathway in genital epithelial cells

**Fig. 3:**
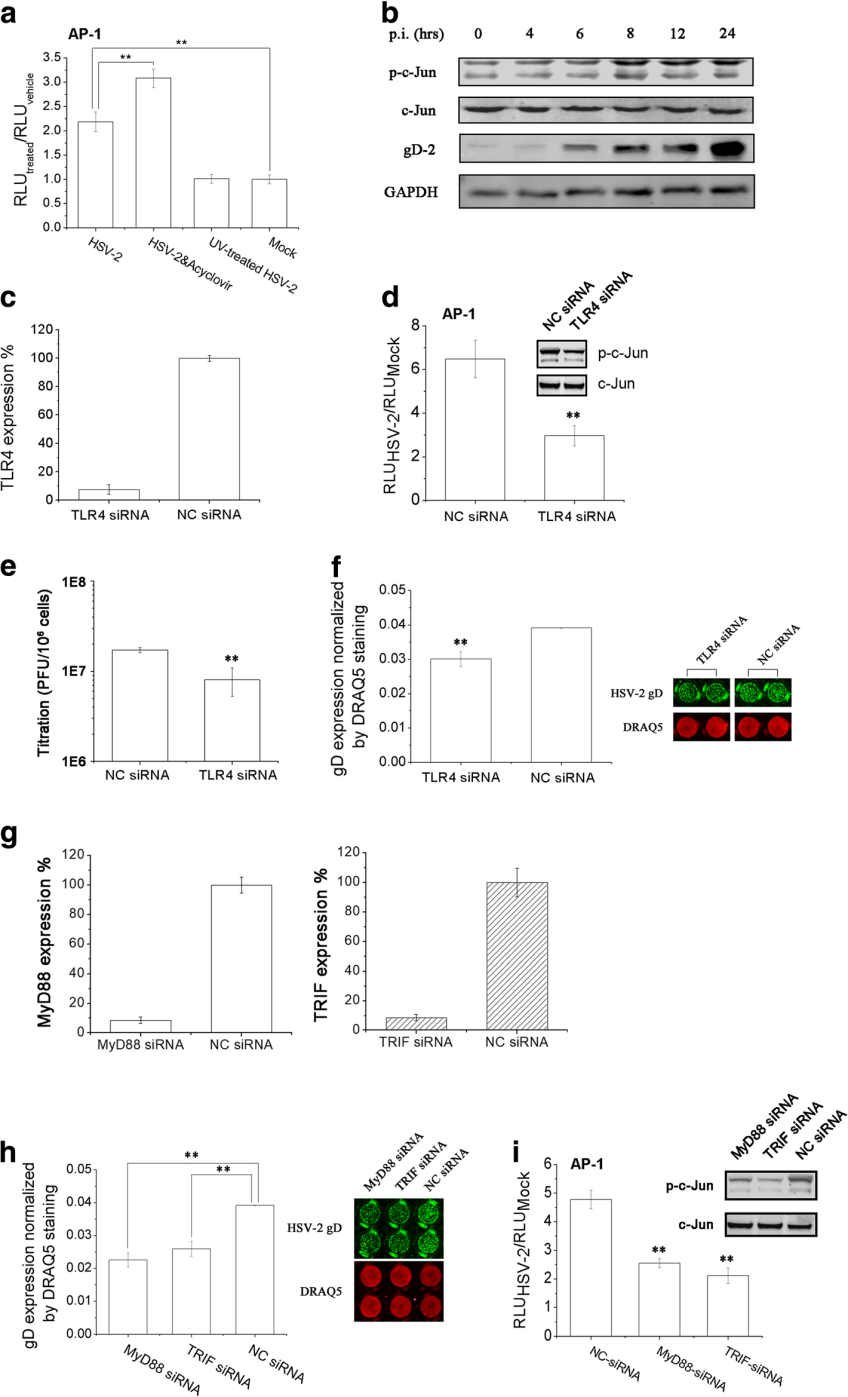
HSV-2 triggered AP-1 transcriptional activation via TLR4-MyD88/TRIF pathway in genital epithelial cells. **a** HSV-2 infection induced AP-1 activation. HEC-1-A cells were co-transfected with pAP-1-luc and pRL-TK. After 24 h, cells were mock-treated or treated with acyclovir (5 μg/ml), and then infected with HSV-2 or UV-treated HSV-2. RLUs were determined as described. **b** HSV-2 could induce c-Jun phosphorylation. HEC-1-A cells were infected with HSV-2 (moi = 1), and at indicated time point, cells were harvested and lysed. c-Jun and phosphorylated c-Jun were determined via western blot. **c** TLR4 specific siRNA knockdown efficiency determination. HEC-1-A cells were transfected with TLR4-specific siRNA oligonucleotide, and the efficiency of TLR4 knockdown was evaluated after 24 h via real-time PCR. **d** Knockdown of TLR4 could attenuate HSV-2-induced AP-1-driven transcription activity and phosphorylation of c-Jun. For AP-1 activity assay, HEC-1-A cells were co-tranfected with TLR4-specific siRNA oligonucleotide or negative control (NC), pAP-1-luc and pRL-TK plasmids. Cells were mock-infected or infected with HSV-2 (moi = 1) 24 h post-transfection. RLUs were determined as described. For p-c-Jun detection, HEC-1-A cells were transfected with TLR4-specific siRNA oligonucleotide or NC. And after 24 h, cells were mock-infected or infected with HSV-2 (moi = 1). c-Jun and phosphorylated c-Jun were determined via western blot 24 h p.i. **e**–**f** Knockdown of TLR4 could impede HSV-2 virions production. HEC-1-A cells transfected with TLR4 siRNA or NC siRNA. After HSV-2 infection for 24 h, cells were freezing and thawing, and the released virions were titrated in a Vero-ICP10P luciferase reporter system (**e**). Or HSV-2 gD expression was evaluated via In-cell Western as described (**f**). **g** MyD88 and TRIF specific siRNAs knockdown efficiency determination. The approach was the same as that described in (**c**). **h**–**i** Knockdown of MyD88 or TRIF expression could attenuate HSV-2-induced AP-1-driven transcription activity and phosphorylation of c-Jun (**h**), and impede HSV-2 replication (**i**). The approach was the same as that described in (**d** and **f**). All experiments were performed three times, and the representative results were shown

In our previous study, we validated that HSV infection was able to induce MAPK activation, especially stimulate p38 and JNK pathway [18]. Firstly, we examined the effect of HSV-2 infection on cellular AP-1 activation in genital epithelial cells. AP-1 could be activated by HSV-2, and not UV-treated HSV-2, demonstrating that virus entry or post-entry steps of viral lifecycle might be essential for AP-1 activation. However, acyclovir treatment could not impede AP-1 activation, implicating that certain viral post-entry events mainly contributed to this effect (Fig. 3a). To rule out the possibility of LPS contamination in HSV-2 virus stock to activate AP-1, high concentration of LPS (20 μg/ml) was exposure to HEC-1-A cells transfected with AP-1-luc plasmid, and no response was observed (Data not shown, HEC-1-A is not sensitive for ~ng/ml LPS exposure, possible reason is that HEC-1-A did not express CD14 molecules), showing that HSV-2 infection might be the main stimuli for AP-1 activation. AP-1 was a heterodimer composed with c-Jun and c-Fos. So we also examined one of the monomer, c-Jun and its phosphorylation level after HSV-2 infection via western blot. As shown in Fig. 3b, HSV-2 infection could induce c-Jun phosphorylation, which was parallel with the results displayed in Fig. 3a.

After that, we considered whether TLR4 expression was necessary for virus-induced AP-1 activation in genital epithelial cells. TLR4-specific siRNA was employed to knockdown its expression in HEC-1-A cells and the results showed that the knockdown efficiency was over 90% and would be then employed in the next experiments (Fig. 3c). We evaluated the effect of TLR4-specific siRNA transfection on HSV-2-induced AP-1 activation and the phosphorylation level of c-Jun, and illustrated that knockdown of TLR4 would attenuate virus-mediated AP-1 activation and c-Jun phosphorylation significantly, concluding that TLR4 might play a role in viral-mediated AP-1 activation pathways (Fig. 3d). Whether silencing TLR4 would impede HSV-2 infection in HEC-1-A cells was examined via quantifying HSV-2 infectious virions. As shown in Fig. 3e, knockdown of TLR4 expression would moderately impede virions production. HSV-2 gD is a kind of viral late gene products which could represent viral replication efficiency [18, 20]. So its effect on HSV-2 gD expression was evaluated via In-cell western and the results was in parallel (Fig. 3f). It was concluded that TLR4 signaling might be associated with viral replication in human genital epithelial cells.

Myeloid differentiation factor 88 (MyD88) and Toll/IL-1R domain-containing adaptor inducing interferon-β factor (TRIF) are two important adaptors for TLR4 to activate downstream AP-1 pathways [21]. We would validate whether HSV-2-induced AP-1 activation was dependent on MyD88/TRIF. Also firstly, we validated the knockdown efficiency of the specific siRNA on MyD88 and TRIF in our culture systems, which were both more than 90% knockdown efficiency (Fig. 3g). Either knockdown of MyD88 or TRIF could suppress HSV-2-mediated AP-1 activation significantly (Fig. 3h). Similarly, knockdown of MyD88 and TRIF would also attenuate HSV-2 gD expression and viral replication (Fig. 3i). It was concluded that HSV-2-mediated AP-1 activation was driven via TLR4-MyD88/TRIF pathway, and also HSV-2 replication might be partly dependent on this axis.

### MD2 is essential for TLR4-mediated AP-1 activation induced by HSV-2 infection

**Fig. 4:**
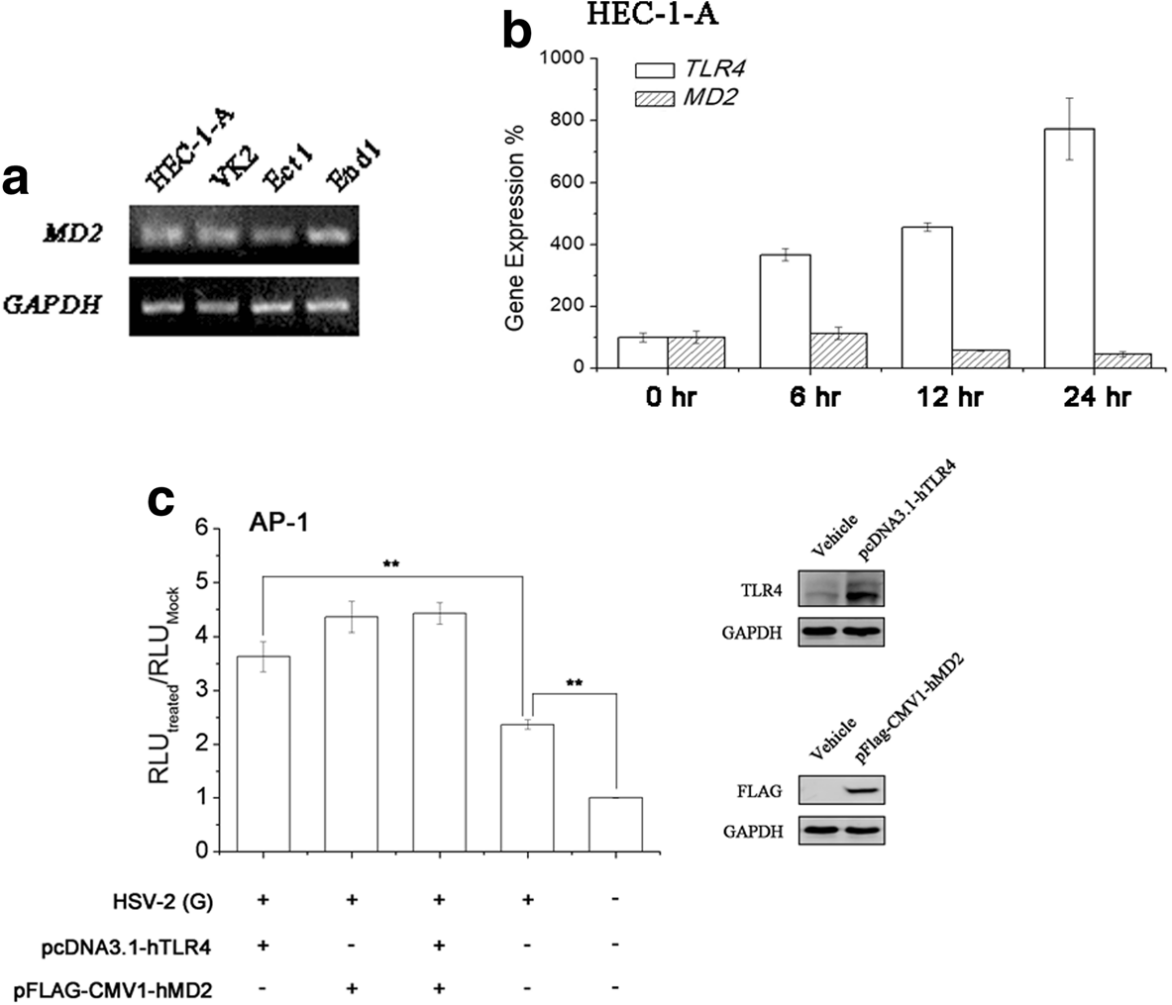
Overexpression of TLR4, MD2 or both augmented HSV-2-mediated AP-1 activation in genital epithelial cells. **a** MD2 expression was determined in 4 different genital epithelial cell lines via RT-PCR. **b** HSV-2 infection did not affect MD2 mRNA expression in HEC-1-A cells (**a**). Total RNA was extracted from HEC-1-A cells at time points as described. TLR4 or MD2 mRNA transcription level was determined through real-time PCR. **c** HEC-1-A cells were co-tranfected with TLR4, MD2 or both expression vectors, and pAP-1-luc and pRL-TK plasmids. Cells were mock-infected or infected with HSV-2 (moi = 1) 24 h post-transfection. RLUs were determined as described. All experiments were performed three times. The representative results were shown

Typically, myeloid differentiation protein 2 (MD2) is reported as the accessory protein for TLR4 mediating endotoxin/lipopolysaccharide (LPS) signaling. Then whether MD2 was involved in TLR4-mediated virus-induced AP-1 signaling in human genital epithelial cells was estimated. As shown in Fig. 4a, it was validated that 4 different kinds of genital epithelial cells from different anatomical positions used in this study constitutively expressed MD2 mRNA. We also examined whether HSV-2 infection could modulate MD2 expression in genital epithelial cells. The result was that unlike TLR4, MD2 expression was not up-regulated during the viral infection (Fig. 4b). The similar results were also obtained in VK2 cells (data not shown). Moreover, the overexpression of TLR4, MD2 or both in HEC-1-A cells could enhance HSV-2-induced AP-1 activation (Fig. 4c), demonstrating that TLR4/MD2 complex was necessary for virus-mediated downstream pathway activation.

### HSV-2 infection increases epithelial cell membrane-associated TLR4 anchoring

**Fig. 5:**
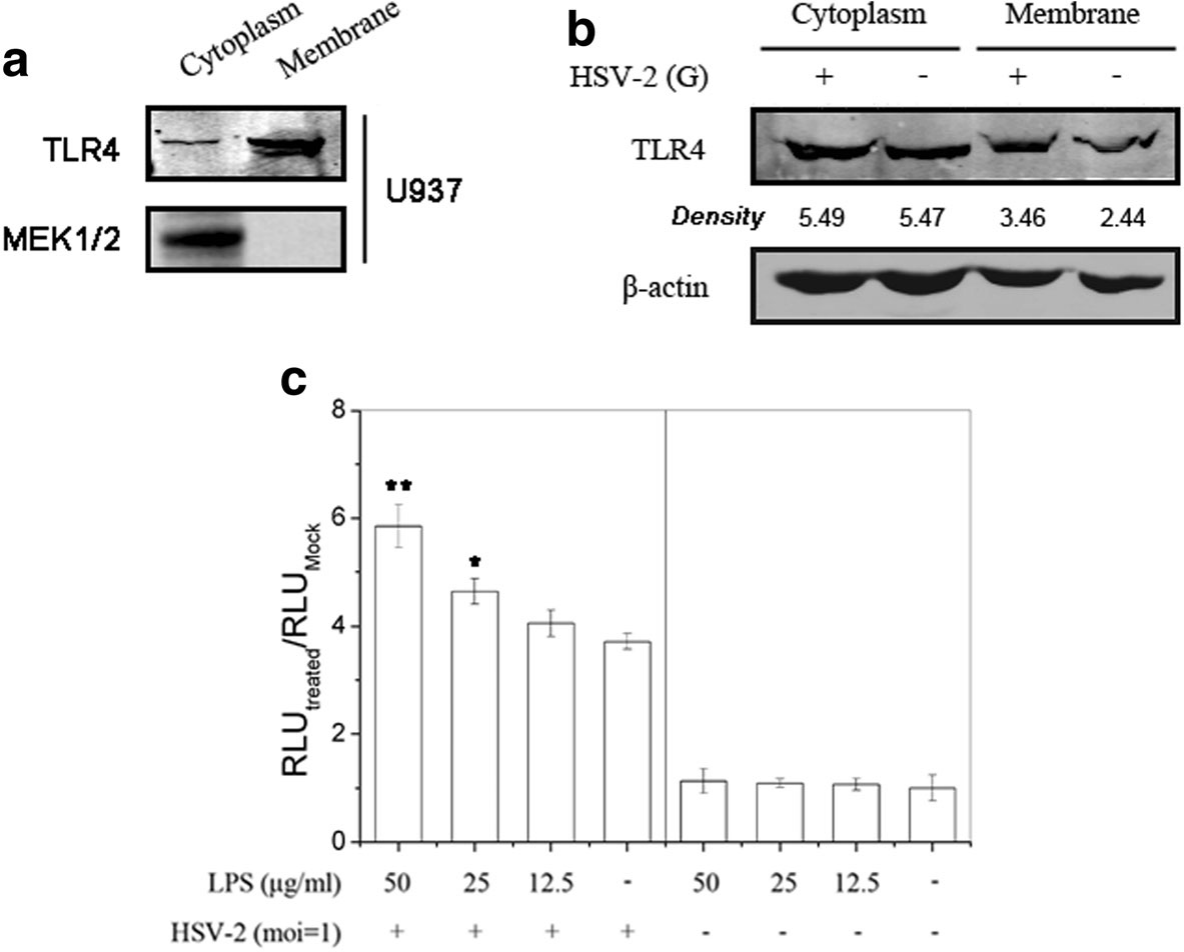
HSV-2 infection up-regulated cell membrane-associated TLR4 expression. **a** The distribution of TLR4 in cytoplasm and membrane of U937 cells. U937 cells were harvested and cytoplasmic or membrane-associated total proteins were exacted as described. TLR4 expression in each component was determined via western blot. MEK1/2 was set as the control for monitoring cytoplasm/membrane fractionation quality. **b** HSV-2 infection increased membrane-anchoring TLR4 content. HEC-1-A cells were mock-infected or infected with HSV-2 (moi = 1). Cells were harvested and cytoplasmic or membrane-associated total proteins were exacted as describe 24 h p.i. TLR4 expressions were examined via western blot. **c** LPS could enhance HSV-2-induced AP-1 activation. HEC-1-A cells were transfected with AP-1-luc plasmid. Cells were mock-infected or infected with HSV-2 (moi = 1) in the absence or presence of serial concentrations of LPS 24 h post-tranfection. Relative luminescence units were determined as described. All experiments were performed twice, and the representative results were shown

Functional TLR4 are located on the surface of cell membrane for sensing extracellular stimuli, such as bacteria LPS. We then extracted total proteins from the membrane or cytoplasm of U937 cells (human leukemic monocyte lymphoma cell line) and HEC-1-A cells, and examined the TLR4 expression localization. The result showed that almost all of TLR4 molecules are expressed in the cell membrane component, and a small amount of TLR4 molecules are localized in cytoplasm in U937 cells, which represented the typical immune cell (Fig. 5a). We also validated cytoplasm/membrane fractionation efficiency via detecting MEK1/2, which was marker of cytoplasm protein. As shown in Fig. 5a, the extraction approach was reliable to separate cytoplasm/membrane proteins. TLR4 molecules were both distributed in cytoplasm and membrane in HEC-1-A cells (Fig. 5b), implying that TLR4 distribution and innate immune status in monocytes and genital epithelial cells are totally different. Then we also investigated whether HSV-2 infection would change TLR4 localization on subcellular structure level in HEC-1-A cells. As shown in Fig. 5b, HSV-2 could augment membrane-associated TLR4 localization, but had less effect on cytoplasmic TLR4. We concluded that this effect would cause genital epithelial cells to transform from static state to immune activation status.

As observed above, HSV-2 infection could change the distribution of TLR4 molecules in HEC-1-A cells, which might activate immune response for genital epithelial cells. To verify this, we employed TLR4 ligand, LPS to activate AP-1 activation in HEC-1-A cells. As shown in Fig. 5c, LPS showed no effect on AP-1 activation in uninfected cells, but it can enhance AP-1-driven transcription activation in HSV-2 infected cells. It came to the conclusion that HSV-2 infection in HEC-1-A cells could increase TLR4 expression on the surface of cell membrane, which might cause enhancement of TLR4-mediated AP-1 activation.

### HSV-2 ICP0 augments AP-1 transcriptional activity

**Fig. 6:**
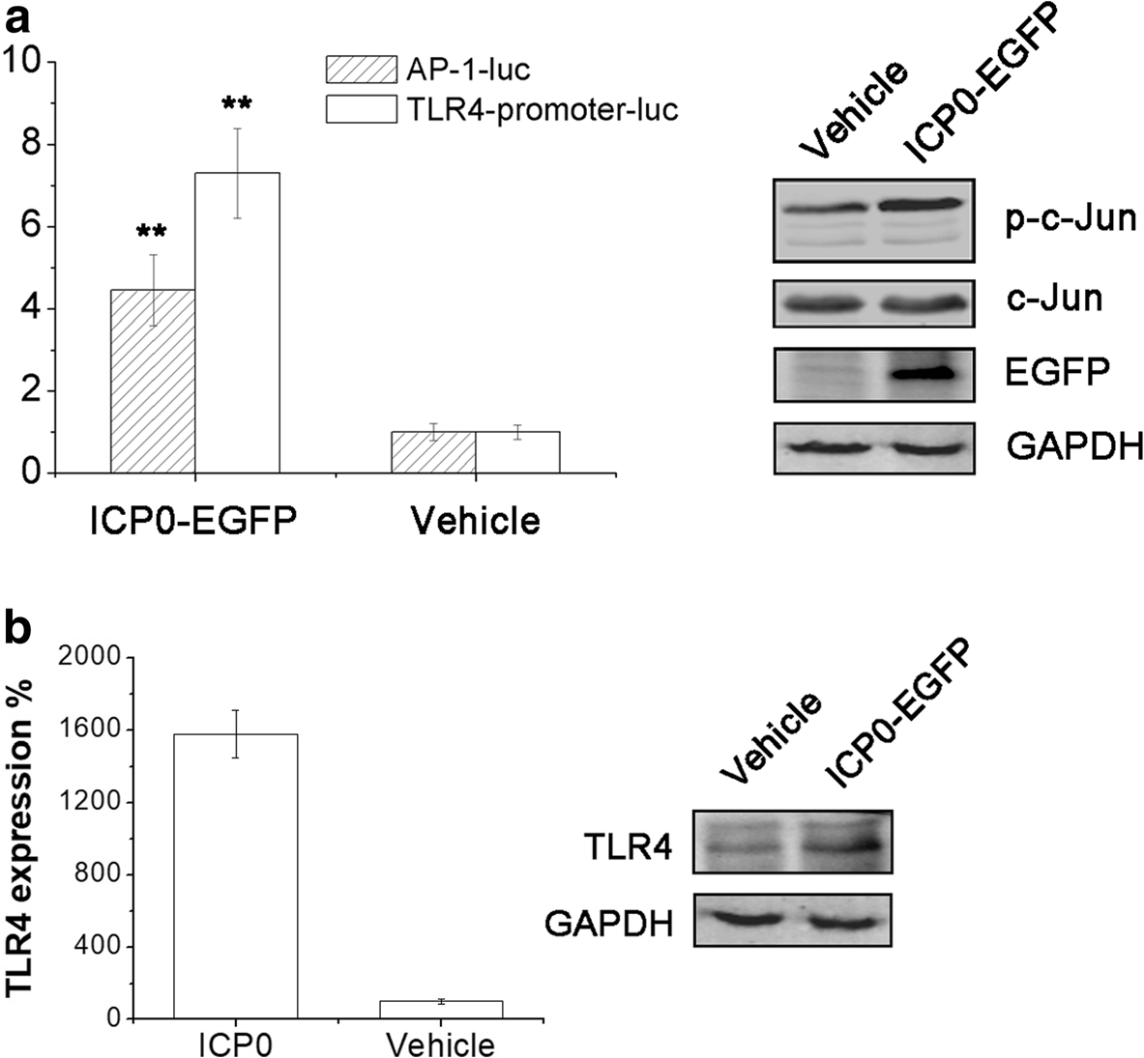
HSV-2 ICP0 induced AP-1 transcriptional and TLR4 promoter activity. **a** ICP0 could enhance TLR4-promoter transcriptional activation, cellular AP-1 activation and c-Jun phosphorylation. For AP-1 activity assay, HEC-1-A cells were co-transfected with AP-1-luc plasmid, and HSV-2 ICP0 expression or vehicle plasmid. Cells were lysed and RLUs were determined as described after 24 h. For TLR4 promoter activity assay, HEC-1-A cells were co-transfected with TLR4-promoter-luc, and HSV-2 ICP0 expression or vehicle plasmid. And the RLUs were determined 24 h post-transfection. For detecting c-Jun phosphorylation, HEC-1-A cells were transfected with vehicle or HSV-2 ICP0 expression plasmid. After 24 h, cells were harvested. c-Jun, phosphorylated c-Jun and EGFP were determined via western blot. **b** Overexpression of ICP0 could up-regulate TLR4 expression. HEC-1-A cells were transfected with vehicle or HSV-2 ICP0 expression plasmid. To evaluate mRNA expression level, total RNA was extracted after 24 h, and TLR4 mRNA transcription level was determined through real-time PCR. To quantify TLR4 protein level, cells were harvested 24 h post-transfection, and TLR4 expressions were examined via western blot. All experiments were performed three times, and the representative results were shown

The previous data exhibited that acyclovir treatment could enhance not only AP-1 transcriptional activity, but also TLR4 promoter activity, implicating that certain virus IE or E gene products might be as the trigger for this effect. After that, we found that overexpression of ICP0 in HEC-1-A cells could induce AP-1-driven transcriptional and TLR4-promoter activation significantly (Fig. 6a–b). Its effect on c-Jun phosphorylation was also investigated via western blot. And the result showed that overexpression of HSV-2 ICP0 would increase c-Jun phosphorylation level (Fig. 6a). TLR4 expression on mRNA and protein levels were also investigated. As shown in Fig. 6c, overexpression of ICP0 could enhance TLR4 protein and mRNA expression in HEC-1-A cells. These findings fitted our previous experiments. We hypothesized that HSV-2 firstly entry into host cells, and then initiated ICP0 expression which stimulated AP-1 activation and TLR4 promoter transcription.

### AP-1 might be essential for TLR4 promoter transcriptional activity

**Fig. 7:**
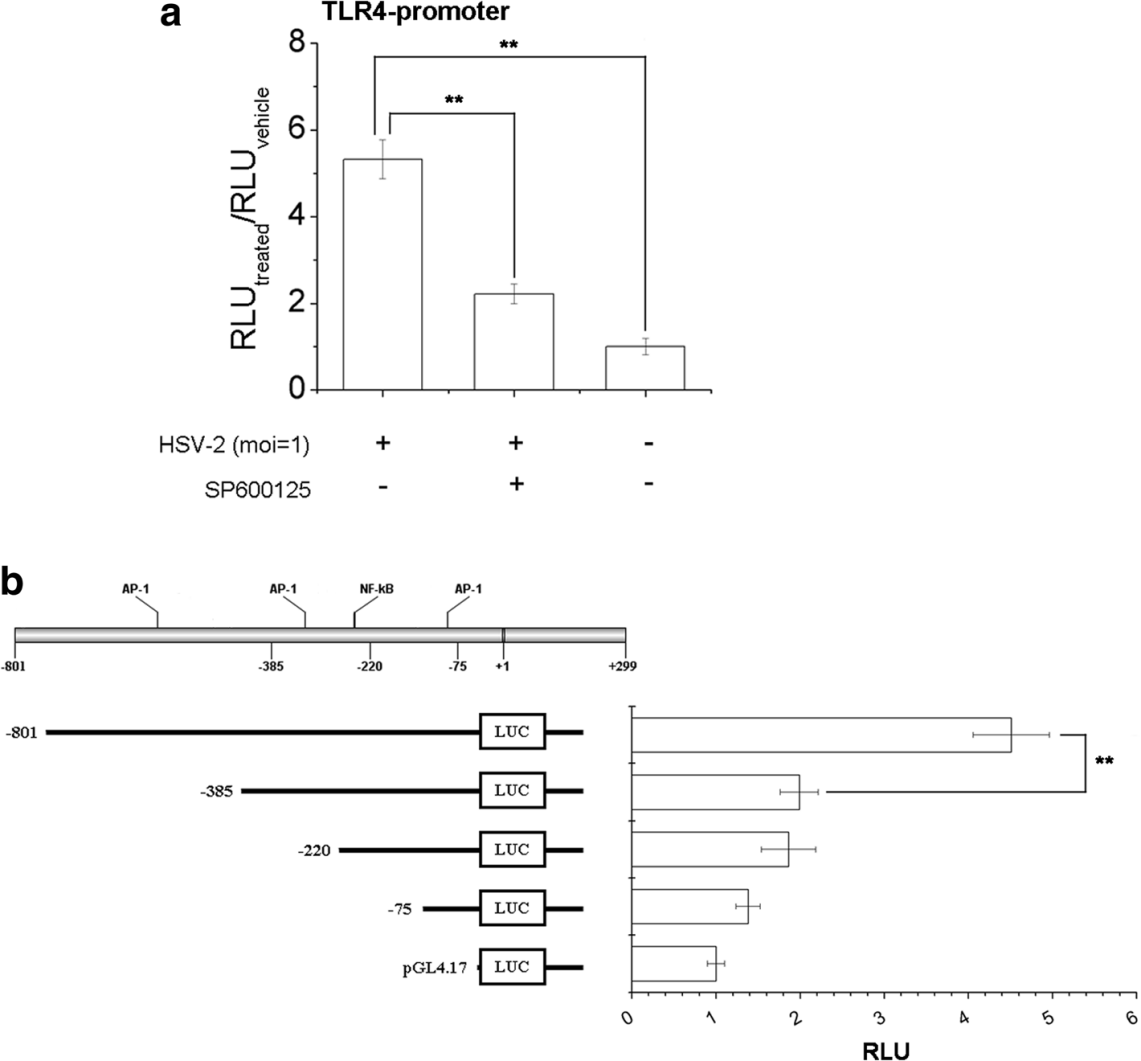
Cellular AP-1 activation might be essential for TLR4 promoter-driven transcription. **a** SP600125 could inhibit HSV-2-induced AP-1 activation. HEC-1-A cells were transfected with TLR4-promoter-luc plasmid. Cells were mock-treated or treated with SP600125 (10 μM), and then infected with HSV-2 (moi = 1) 24 h post-transfection. The relative luminescence units were determined 24 h p.i. **b** AP-1 transcriptional factor binding site was important for TLR4 promoter activity. HEC-1-A cells were transfected with TLR4-promoter-luc, and its truncated promoter luciferase reporter plasmid. After 24 h, cells were infected with HSV-2 (moi = 1) and the relative luminescence units were determined 24 h p.i. All experiments were performed three times, and the representative results were shown

To investigate the relationship between AP-1 and TLR4 expression regulation, a potent specific JNK inhibitor, SP600225 was chosen to evaluate its inhibitory effect on HSV-2-induced TLR4 promoter activation. As shown in Fig. 7a, SP600125 could inhibit virus-mediated TLR4 promoter activation significantly, implicating that JNK and its downstream AP-1 was important for the initiation of TLR4 expression in genital epithelial cells.

Further, we employed Alibaba 2.1 online software to predict transcriptional factors binding sites in TLR4 promoter, and AP-1 and NF-κB binding sites were labeled in TLR4 promoter schematic in Fig. 7b. We then constructed some truncated TLR4-promoter luciferase reporter plasmid to evaluate the contribution of these predicted transcriptional factor binding sites to TLR4 promoter activation. The results demonstrated that predicted AP-1 binding site (− 566~ − 556) might play an important role in AP-1-driven TLR4-promoter activation, thus up-regulated TLR4 expression (Fig. 7b). Other AP-1 and NF-κB binding sites did not exhibit any significant effect on it. In conclusion, AP-1 might be essential for TLR4-promoter activation and its expression modulation.

## Discussion

Human genital epithelial cells are primary physiologic barrier against pathogenic microorganisms which would cause sexual transmitted diseases. Several studies have indicated that certain pathogens could induce innate immune responses via pattern recognition receptors PPRs in genital epithelium, and these effects would activate anti-viral or anti-bacterial responses. But in some cases, some pathogens would hijack cellular innate immune systems to facilitate their sexual transmission and infection [10, 11]. Compared with immune cells, genital epithelial cells have a distinct innate immune system, and would trigger a unique immune response to sense and resist infection. TLRs are the most studied and best characterized PPRs, which are identified as fundamental components for the innate immune response to bacterial or viral pathogens in epithelial cells.

Recent evidence showed that herpes simplex virus could trigger cellular PPRs to modulate innate immune defenses. Triantafilou et al. reported that HSV-2 could induce vaginal cells activation via TLR2, TLR9 and DNA sensors DAI and IFI16 [22]. And TLR2 has also been reported to be vital in HSV sensing, which was triggered by the interaction between HSV virion glycoproteins and TLR2 [16, 23, 24]. However, fewer studies focused on the other PPRs interaction with HSV-2. Recently, Liu et al. demonstrated that TLR4 could sense HSV-2 infection human cervical epithelial cells to induce NF-κB-driven transcription activity, and HSV-2 would also up-regulate TLR4 expression [17]. However, the precise mechanism of virus-induced TLR4 expression up-regulation was unclear. In this study, we identified TLRs expression profiles in human genital epithelial cells and their expression regulation by HSV-2 infection comprehensively, and the results exhibited that TLR4 was highly expressed in two genital epithelial cells and would be up-regulated significantly by HSV-2 infection. Further studies illustrated that TLR4 contributes to HSV-2-induced AP-1 activation, and AP-1 might play an important role in TLR4-promoter activation. We concluded that TLR4 might be a significant sensor for response herpes virus infection in human genital epithelium.

It was hypothesized that some transcriptional factors activity was necessary for TLR4 promoter-driven transcription initiation. We have predicted some transcriptional factors binding sites in TLR4 promoter region, and found that an AP-1 binding site (− 566~ − 556) was more important through truncated promoter scanning assay. These data were reasonable to the conclusion that virus-induced AP-1 activation modulated TLR4 expression and feed back to HSV-2 infection sensing. The detailed regulation mechanisms should be further studied.

In general, TLR4 was the major extracellular receptor to sense LPS derived from Gram-negative bacteria to induce downstream critical proinflammatory responses. Much evidence have proved that beside of bacteria components, certain virus or virus-associated proteins could be as potential inducer for TLR4-mediated downstream signal pathway activation. Machida et al. illustrated that hepatitis C virus infection and replication induced TLR4 expression and enhanced TLR4-mediated IFN-β and IL-6 production [25]. Respiratory syncytial virus was most well-known virus which could initiate innate immune responses via TLR4 [26–28]. Del et al. also reported that host TLR4 interacted with HIV-1 gp120, leading to intracellular pathways and biologic activities that mediate proinflammatory and profibrogenic signals [29]. In this study, it was demonstrated that HSV-2 could activate intracellular AP-1-driven transcription via TLR4-MyD88/TRIF axis, and this effect might depend on viral IE and E gene expression accumulation. Further studies implicated that HSV-2 ICP0 might be the key factor for AP-1 activation. ICP0 is an important IE regulatory protein of HSV that play vital roles in viral replication, cell growth and apoptosis [30, 31]. It is believed to be able to activate transcription, not only viral genes but also host ones. Diao et al. reported that HSV-1 ICP0 could strongly activate AP-1 responsive genes specifically via JNK pathway activation [32], which illuminated us that HSV-2 ICP0 accumulation at early stage of infection causing AP-1 activation might be the reason for TLR4 promoter activation. And further studies primarily proved that AP-1 activation was associated with TLR4 expression regulation. It was concluded that ICP0 accumulation stimulated host AP-1 activation, and then mediated TLR4 promoter activation, which would consistently activate TLR4 sensing HSV-2 infection. Previously, HSV-2 ICP10PK was reported to modulate AP-1, MEK/MAPK and JNK/c-Jun [33, 34]. Whether HSV-2 ICP10PK expression would influence TLR4 expression will be studied further. Additionally, intracellular TLR4 would be activated by intracellular LPS in intestinal epithelial cells [35]. It is hypothesized that certain HSV viral proteins would be recognized by intracellular Golgi bodies-localized TLR4, and activate downstream signaling pathways, which will be also studied further.

The accessory protein MD2 has been implicated in LPS-mediated activation of the innate immune system by functioning as a co-receptor with TLR4 for LPS binding at the cell surface [36]. We have also proved that overexpression of MD2 in genital epithelial cells enhanced HSV-2-mediated AP-1 activation, demonstrating that TLR4-MD2 complex are necessary for sensing HSV-2 infection, similar with that of LPS. However, MD2 expression was stable during the HSV-2 infection (data not shown). The precise mechanisms should be investigated further.

Membrane-anchored TLR4 molecules are necessary as the functional receptor for sensing exogenous stimuli. We examined the TLR4 distribution in genital epithelial cells, and results illustrated that a large amount of TLR4 existed in cytoplasm, not on the surface of membrane, which was opposite to that in monocytes. Thus, these two cell types displayed the distinct innate immune status responding to LPS and other stimuli (data not shown). Although HSV-2 had ability to up-regulated TLR4 expression, we did not understand whether this effect would change TLR4 function or innate immune status in genital epithelial cells. After that, TLR4 expression in cytoplasm and membrane was determined, and the results showed that viral infection up-regulated membrane-associated TLR4 moderately. We hypothesized that HSV-2 replication could induce TLR4 expression and promote TLR4 translocation from cytoplasm to cell membrane, which cause TLR4-mediated cascade signal amplification and downstream AP-1 activation. This effect might change innate immune balance and influence resistance of epithelial cell against other bacterial pathogens.

## Conclusions

In summary, our findings firstly revealed that TLR4 played a vital role in sensing HSV-2 infection in human genital epithelial cells, and TLR4-MyD88/TRIF-AP-1 pathway is essential for HSV-2-induced up-regulation of TLR4 expression, which implicated that TLR4 could be as a virus sensor for herpes infection.
